# The complete mitochondrial genome of Steppe Whiskered Bat (*Myotis aurascens*; Kuzyakin, 1935) and phylogenetic analysis

**DOI:** 10.1080/23802359.2022.2059408

**Published:** 2022-04-04

**Authors:** Xiufeng Yang, Qi Wang, Lei Zhang, Saru Bao, Shihu Zhao, Huashan Dou, Honghai Zhang

**Affiliations:** a College of Life Science, Qufu Normal University, Qufu, PR China; b Hulunbuir Academy of Inland Lakes in Northern Cold & Arid Areas, Hulunbuir, PR China

**Keywords:** *Myotis aurascens*, phylogenetic analysis, mitochondrial genome

## Abstract

In this study, the complete mitochondrial genome of Steppe Whiskered Bat was sequenced for the first time using muscular tissue. The whole mitochondrial genome was 16,771 bp in length, consisting of two ribosomal RNA genes, 13 protein-coding genes, 22 transfer RNA genes, and one control region (D-loop). Phylogenetic analysis using PAUP based on mitochondrial genome (12 PCGs, except *ND6*) of 16 other Vespertilionidae species revealed the close relationship of *M. aurascens* with other related *Myotis* species.

*Myotis aurascens* (Steppe Whiskered Bat; Kuzyakin, 1935; Mammalia: Chiroptera: Vespertilionidae) distributed in south-east Mediterranean and extended eastwards out of the region into steppe Europe, and south-west Asia. Due to cryptic characteristics with *M. mystacinus* and *M. ikonnikovi*, the exact population size is not known (Benda 2004). In this study, the sample of *M. aurascens* was collected through field survey in Hulun Lake National Nature Reserve, Inner Mongolia, China (48°54′28.41″, 117°5′2.65″E). The sample was frozen in ultra-low temperature freezer and stored in the Animal Specimen Museum of Qufu Normal University, Qufu, Shandong, China (collector: Xiufeng Yang, yangxf9066@163.com), and the accession number was QFA20180059. The DNA was extracted with the DNeasy Blood & Tissue kit (QIAGEN, Beverly, MA).

The sample was sequenced in an Illumina MiSeq platform with 150 PE. All sampling procedures and experimental manipulations held the proper permits (2022002). After manual assembling and annotating using online software Banklt, the genome was deposited in GenBank with the accession number OK053029.

The complete mitochondrial genome of *M. aurascens* is a closed circle with a length of 16,771 bp. It contains 37 genes, including two ribosomal RNA genes (rRNAs), 13 protein-coding genes (PCGs), and 22 transfer RNA genes (tRNAs), which was similar to other species of the same genus (Jebb et al. 2017). The light strand (L-strand) contains eight tRNA (*tRNA^Gln^*, *tRNA^Ala^*, *tRNA^Asn^*, *tRNA^Cys^*, *tRNA^Tyr^*, *tRNA^Ser^*, *tRNA^Glu^*, and *tRNA^Pro^*) and one PCGs (*ND6*), and other genes are located in the heavy strand (H-strand). The overall base composition of *M. aurascens* was estimated to be A: 33.9%, T: 30.9%, C: 22.2%, and G: 13.0%, and the higher content of A + T (64.8%) than that of C + G (45.2%). The gene structure, content, and arrangement were found to be similar to other *Myotis* species reported previously (Chung et al. 2018).

To validate the phylogenetic position of *M. aurascens*, 16 species (the *Tadarida latouchei* was chosen as an out-group) of Vespertilionidae’ 12 mitochondrial protein coding genes (except *ND6*) were selected to construct maximum-likelihood (ML) tree by PAUP 4.0b10 (Swofford 2002). According to the AIC criterion, GTR + I+G was selected as the best-fitting nucleotide substitution model using MrModeltest 3.7 (Posada 2005). Amino acid sequences from each PCG were aligned by MEGA11 (Koichiro et al. 2021). The result of Phylogenetic tree showed that *M. aurascens* was close to other *Myotis* species (Figure 1). In addition, the *Myotis* was closely related to the *Murina* in the Vespertilionidae, which was also supported by previous study (Platt et al. 2018). We expected the data of present study to be useful for further research and phylogenetic relationship of Vespertilionidae. The data of our study are vital for the further researching Vespertilionidae and their phylogenetic relationship.

**Figure 1.**
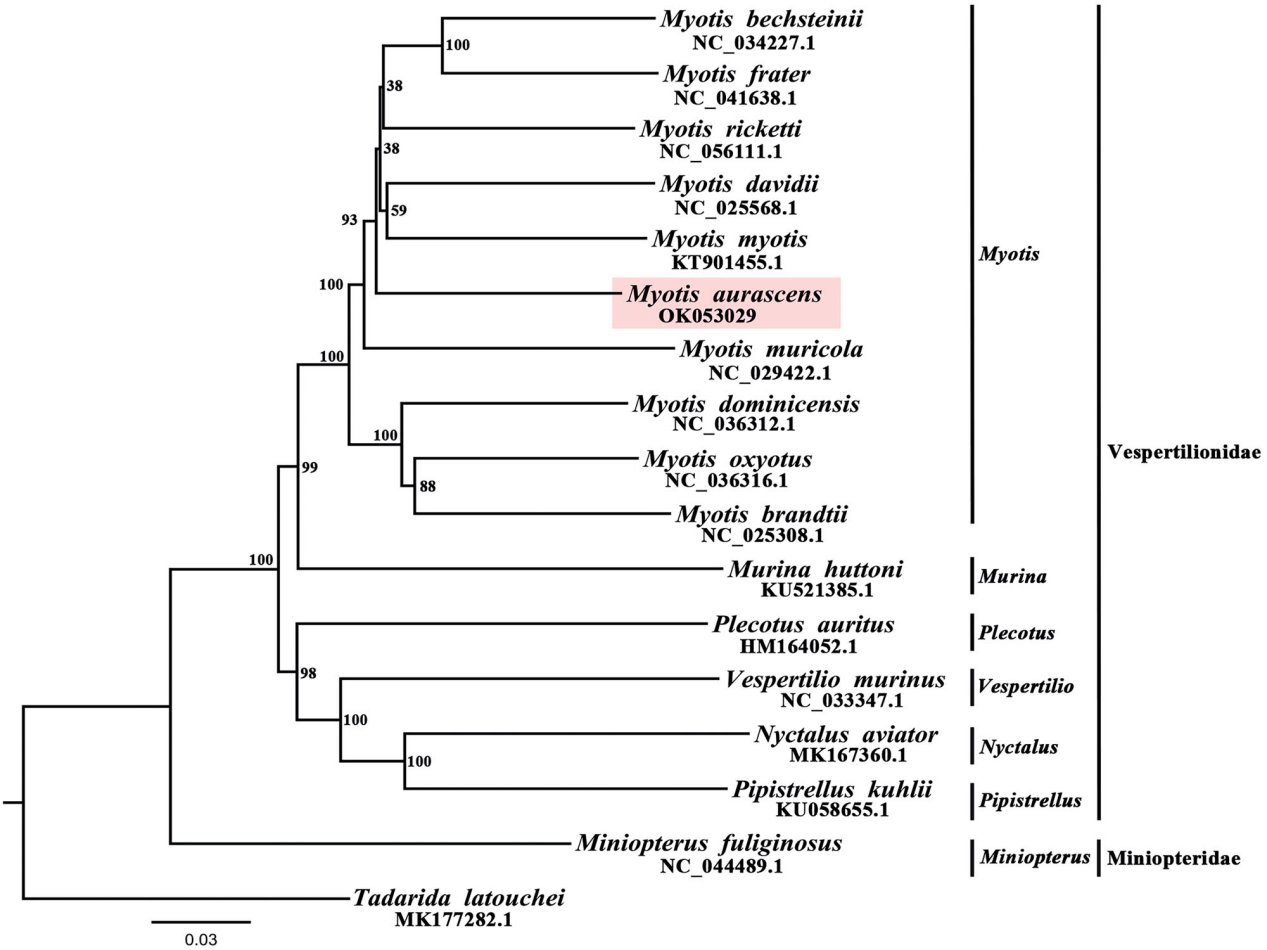
Maximum-likelihood (ML) trees of 17 species based on 12 protein coding genes (except *ND6*). The accession numbers of species which were downloaded from GenBank and probabilities display in the nodes.

## Authors contributions

Yang XF and Zhang HH conceived and designed this study; Wang Q, Bao SR, and Dou HS performed the samples collection and DNA extractions; Yang XF, Zhang L, and Zhao SH performed all bioinformatics analyses; Yang XF and Wang Q wrote the drafting of the paper; Zhang HH and Dou HS revised the critically for intellectual content and the final approval of the version to be published; and that all authors agree to be accountable for all aspects of the work.

## Data Availability

The genome sequence data that support the findings of this study are openly available in GenBank of NCBI at https://www.ncbi.nlm.nih.gov/ under the accession no. OK053029. The associated BioProject, SRA and BioSample numbers are PRJNA768968, SRR16214749, and SAMN22072506, respectively.
